# Production, Characterization and Biocompatibility of Marine Collagen Matrices from an Alternative and Sustainable Source: The Sea Urchin *Paracentrotus lividus*

**DOI:** 10.3390/md12094912

**Published:** 2014-09-24

**Authors:** Cristiano Di Benedetto, Alice Barbaglio, Tiziana Martinello, Valentina Alongi, Dario Fassini, Emanuele Cullorà, Marco Patruno, Francesco Bonasoro, Mario Adolfo Barbosa, Maria Daniela Candia Carnevali, Michela Sugni

**Affiliations:** 1 Department of Biosciences, University of Milan, Via Celoria 26, 20133 Milan, Italy; E-Mails: cristiano.dibenedetto@unimi.it (C.D.B.); valentina_alongi88@libero.it (V.A.); dario.fassini@gmail.com (D.F.); emanuele.cullora@tiscali.it (E.C.); francesco.bonasoro@unimi.it (F.B.); daniela.candia@unimi.it (M.D.C.C.); michela.sugni@unimi.it (M.S.); 2 Department of Comparative Biomedicine and Food Science, University of Padova, Agripolis Viale dell’Università 16, 35020 Legnaro (PD), Italy; E-Mails: tiziana.martinello@unipd.it (T.M.); marco.pat@unipd.it (M.P.); 3 INEB-Institute of Biomedical Engineering, University of Porto, Rua do Campo Alegre, 823, 4150-180 Porto, Portugal; E-Mail: mbarbosa@ineb.up.pt

**Keywords:** sea urchin collagen, native fibril, collagen matrix, biocompatibility, tissue regeneration, mesenchimal stromal cells

## Abstract

Collagen has become a key-molecule in cell culture studies and in the tissue engineering field. Industrially, the principal sources of collagen are calf skin and bones which, however, could be associated to risks of serious disease transmission. In fact, collagen derived from alternative and riskless sources is required, and marine organisms are among the safest and recently exploited ones. Sea urchins possess a circular area of soft tissue surrounding the mouth, the peristomial membrane (PM), mainly composed by mammalian-like collagen. The PM of the edible sea urchin *Paracentrotus lividus* therefore represents a potential unexploited collagen source, easily obtainable as a food industry waste product. Our results demonstrate that it is possible to extract native collagen fibrils from the PM and produce suitable substrates for *in vitro* system. The obtained matrices appear as a homogeneous fibrillar network (mean fibril diameter 30–400 nm and mesh < 2 μm) and display remarkable mechanical properties in term of stiffness (146 ± 48 MPa) and viscosity (60.98 ± 52.07 GPa·s). *In vitro* tests with horse pbMSC show a good biocompatibility in terms of overall cell growth. The obtained results indicate that the sea urchin *P. lividus* can be a valuable low-cost collagen source for mechanically resistant biomedical devices.

## 1. Introduction

Collagen is the main structural component of animal tissues and shows peculiar mechanical properties conferring strength and elasticity to the tissue itself. The use of collagen as a biomaterial for scaffold development is a topic of great interest, particularly in the tissue engineering field [1,2].

The most used commercial sources of collagen are calf skin and bones, which, however, could carry the risk of serious disease transmission (*i.e.*, bovine spongiform encephalopathy) [3]. This induced researchers to investigate alternative collagen sources, the most interesting and promising ones coming from marine organisms. Scaffolds made of soluble jellyfish or squid collagen exhibited lower immunogenicity and higher cell viability than other naturally derived biomaterials, including bovine collagen, gelatin, hyaluronic acid and glucan [3,4].

Furthermore, good cell viability and osteo-inductive potential was displayed by scaffolds derived from marine sponges [5]. This latter *in vitro* feature is in agreement with the fact that collagen plays a fundamental role as universal template for *in vivo* skeletogenesis processes in marine invertebrates [6,7].

Collagen derived from fish, as well as from cuttlefish outer skin waste material, is also under evaluation as a promising alternative [8,9].

**Figure 1:**
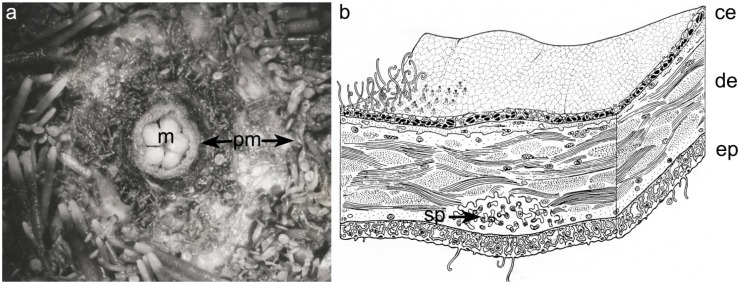
The peristomial membrane of *P. lividus.* (**a**) Oral side of *P. lividus*. Around the mouth (m), the peristomial membrane (pm) is well detectable; (**b**) Schematic anatomy of the peristomial membrane. Dermis (de), ossicles (sp), epidermis (ep), coelomic epithelium (ce). Reproduced with permission from [11]; published by *Ital. J. Zool.*, Journals Taylor & Francis Group, 1995.

Among marine organisms sea urchins are very common coastal inhabitants. They possess a soft membranous area surrounding their mouth: the peristomial membrane (PM) (Figure 1a,b).

The PM mainly consists of a thick ossicle-reinforced dermal layer externally covered by an epidermis and internally by a coelomic epithelium. The collagenous meshwork is the most significant component in terms of both structure and mechanical properties [10,11]. 

The PM collagen is similar to the mammalian type-I in terms of chain composition, immunoreactivity and ultrastructure (D-period) [12,13,14] and therefore may represent a suitable alternative source of collagen to be used for biomedical applications. In this research we specifically focused on the common sea urchin *Paracentrotus lividus*, the main Mediterranean and North-Atlantic edible species [15]. Its gonads are collected for food purpose and the rest of the body, including the PM, is discharged: therefore this waste-material can be potentially exploited for an “eco-friendly” and low-cost collagen extraction. Additionally, sea urchins are harvested in several countries (Japan, USA, France), thus potentially allowing an industrial and constant supply of rough collagenous material to be used by biotech companies.

Differently from most commercial mammalian collagens, sea urchin collagen cannot be extracted by traditional methods of acid solubilization, which normally make it in a hydrolyzed jelly form. On the contrary PM collagen is easily extracted in its insoluble fibrillar form, *i.e.*, the native form. This characteristic provides the considerable advantage of producing scaffolds/substrates more closely mimicking the natural extracellular matrix (ECM) and potentially displaying higher mechanical performances. In tissue engineering it is now well known that mechanical properties and scaffold geometry/ultrastructure play a crucial role in cell differentiation and specification [16,17]. By specifically implementing and adjusting previous protocols [18,19], we were able to isolate undamaged native collagen fibrils from the sea urchin *P. lividus* [20] in a sufficient amount and purity to produce fibrillar scaffolds.

In tissue engineering the assembling of collagen fibrillar matrices usually includes fibrillization steps on partly defibrillated or hydrolized collagen [21,22]. This method causes the production of fibrils which are only partially similar to the native ones in terms of structure and mechanical properties [22]. Among the authors who worked with native collagen fibrils from both vertebrates [23,24,25,26,27,28,29,30,31,32] and invertebrates [18,33,34,35,36,37], only a few, *i.e.*, [30,32]—and exclusively in vertebrates—produced substrates for cell cultures using this type of collagen (in literature often regarded as “insoluble collagen”).

Irrespective of its form (hydrolized or fibrillar), collagen for scaffolding needs appropriate cross-linking procedures for stabilizing the matrix and, if needed, for reducing enzymatic degradation phenomena when surgically implanted *in vivo*. Since a few years ago, glutaraldehyde was one of the most used substances for collagen crosslinking, but it was recently substituted by other compounds due to its potential cell toxicity [38,39]. Among alternative biocompatible cross-linkers, EDC-NHS [1-Ethyl-3-(3-dimethylaminopropyl)-carbodiimide (EDC)/Nhydroxysuccinimide (NHS)] is well known and widely used for its several advantages and for *in vitro* system applications [3,40,41,42,43].

The present work is addressed to evaluate sea urchin collagen as a potential low-cost alternative for the production of scaffolds for biomedical applications. This was achieved following different steps and approaches, including the development of a specific protocol for fibrillar collagen extraction and matrix preparation as well as a deep evaluation of the produced matrices in terms of ultrastructure, mechanical properties (stiffness and viscosity) and *in vitro* biocompatibility.

## 2. Results and Discussion

### 2.1. Collagen Extraction

As previously underlined, most of the common and industrial methods for collagen extraction are based on an acid-solubilization and partial hydrolysis of the collagen triple helix [22], which consequently involves the partial or total loss of structural fibril organization. The method we developed here leads to the extraction of intact collagen fibrils from *P. lividus* PM. 

**Figure 2:**
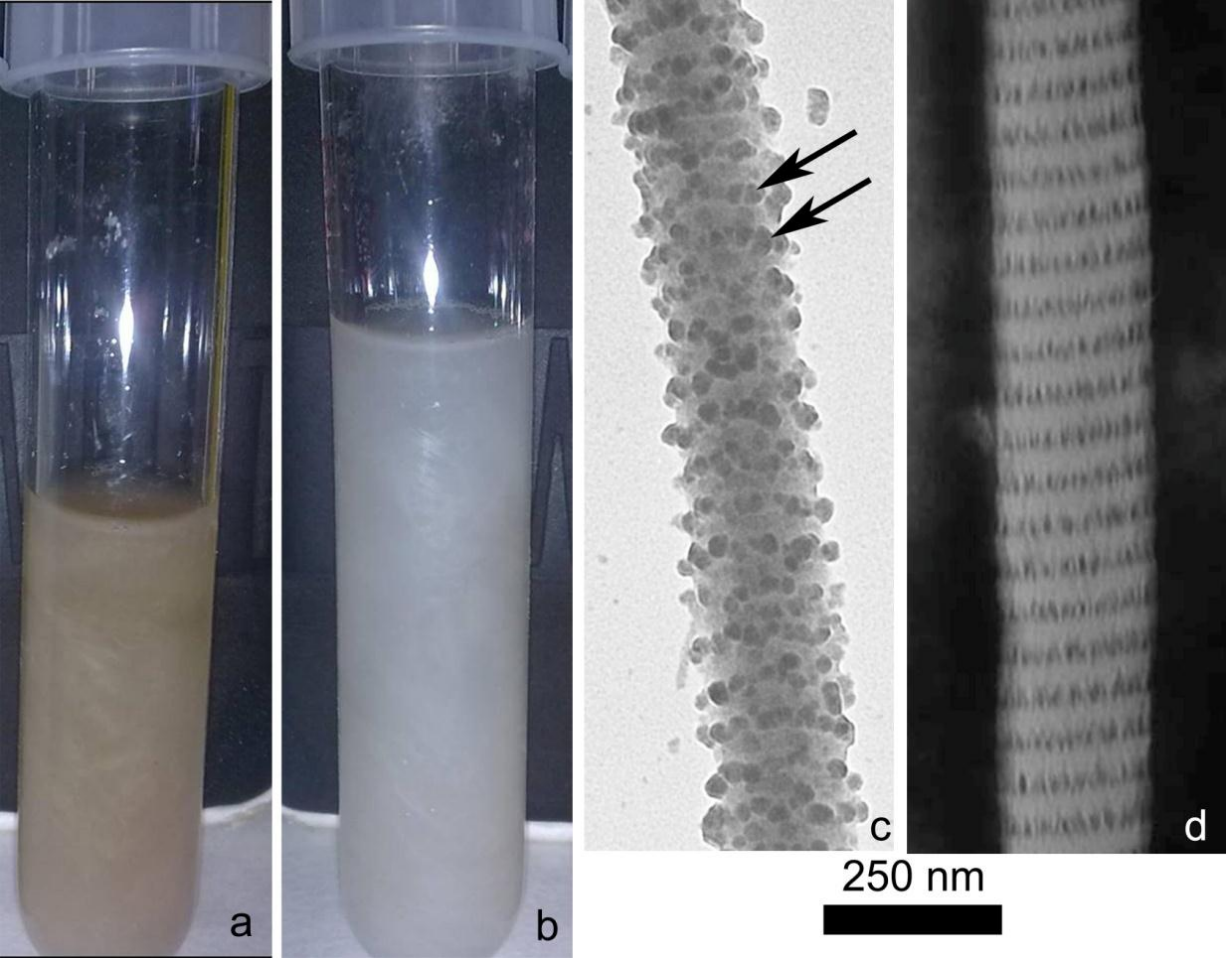
Collagen fibrils. (**a**) Collagen aqueous fibrillar suspension obtained by a protocol in which the hypotonic and decellularizating solutions were omitted. The brown color is mainly due to the presence of cell debris and pigments; (**b**) Clean aqueous collagen fibrillar suspension obtained by the complete protocol; (**c**) Transmission Electron Microscope (TEM). Cuprolinic Blue staining on isolated collagen fibrils. After collagen extraction, fibril-associated glycosaminoglycans (GAGs) could be detected periodically organized along the fibril surface (arrows); (**d**) TEM, negative staining on isolated collagen fibrils. The collagen fibril D period is clearly visible.

Besides the basic collagen matrix, the PM contains other different components (cellular elements, ossicles; Figure 1b), which represent a serious obstacle in obtaining a clean fibril suspension (Figure 2a).

In order to properly remove both cell debris, skeletal parts and pigments, the minced native tissue was sequentially treated with two different specifically developed solutions: a hypotonic solution and a SDS-based decellularizing solution [20,44]. The former induced cell lysis whereas the latter removed cell debris and most of the non-collagenous extracellular material acting as a detergent (Figure 2b). Only the most strictly fibril-associated glycosamynoglycans (GAGs) remained, periodically organized along the fibril surface according to the standard D-patterning (Figure 2c,d). The presence of these GAGs is important to preserve fibril integrity [45], can increase matrix hydrophylicity (GAGs are polianionic molecules) and allows the production of a more biomimetic collagen matrix. Indeed, the ECM naturally contains GAGs and proteoglycans (GAG-protein association), which are important for several functions including tissue hydration, structural organization and cell adhesion. In tissue engineering, GAGs are often secondarily added to improve scaffold structural, mechanical and physiological performances [46].

Careful PBS washing of the minced tissue before the final disaggregation step (in β-mercapto-ethanol solution) resulted a crucial passage: an incomplete removal of SDS strongly reduced the yield of following fibril extraction. On the other hand SDS treatment was clearly necessary for obtaining a clean collagen suspension suitable for scaffold production. 

After 3–4 days in the disaggregating solution a suspension of fibrils could be observed (Figure 2b). 

Undissociated collagen fibers were then removed by a filtration step, although increasing the purity of the extracted fibrils, often implied a partial loss of material and might be problematic due to filter occlusion. The employment of a gradual two-step filtration (200 µm mesh filtering followed by a 100 µm mesh passage) represented the best compromise between the need of obtaining a clean collagen suspension and the effective yield of collagen extraction from the PM. 

**Figure 3:**
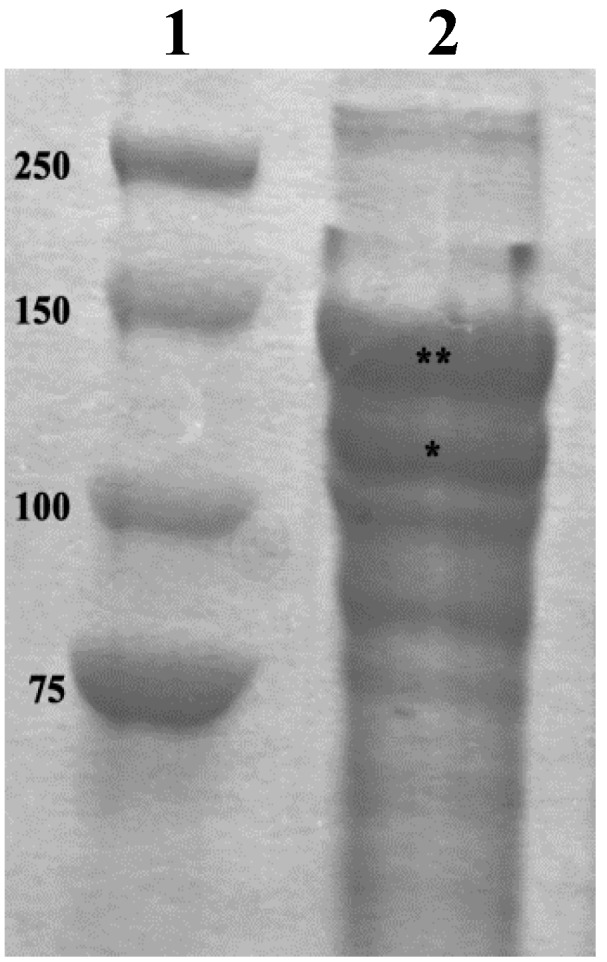
SDS-PAGE gel of the obtained sea urchin collagen suspension. ** = α_1_ collagen chain; * = α_2_ collagen chain. Lane 1 = marker. Lane 2 = collagen suspension.

Removal of the β-mercapto-ethanol (that might be toxic for cells if present in high concentrations) was obtained dialyzing the collagen suspension against EDTA (3 h) and, subsequently, against distilled water (overnight), which represented the final stocking medium. SDS-page analyses (Figure 3) showed that the main proteins contained in the obtained suspension correspond to the α_1_ (140 kDa) and α_2_ (120 kDa) sea urchin type I-like collagen chains previously described in the literature [13,47]. As expected for collagen SDS-page, some of these chains do not completely dissociate under denaturating condition thus producing the characteristic β-sheet bands at 260 kDa (α_1_ + α_2_) and 280 kDa (α_1_ + α_1_). Overall these biochemical data confirm the main collagenous nature of the fibrillar suspension. Further more detailed analyses will help to clarify the nature of the other observed bands (e.g., other collagen types or collagen associated proteoglycans).

The protocol here described represents the most effective technique to obtain a pure and highly concentrated fibril suspension. Collagen yield from the PM was calculated: in optimal conditions and taking into account 7 different collagen stocks produced in different periods of the year, the average yield is 7% (dry weight collagen fibrils/dry weight PM). This is a much higher value if compared to the yield previously obtained in mammals with a similar protocol [24]. The classical acid-based extraction procedures might provide higher yields, but usually implies damages of fibril structural integrity [31].

The obtained aqueous collagen suspension (Figure 2b) can be directly stored at −20 °C for at least 3 months without significant problems in re-suspension as on the contrary occurred with solid collagen stocks (dried at 37 °C in silicone molds).

### 2.2. Collagen Matrix Production 

**Figure 4:**
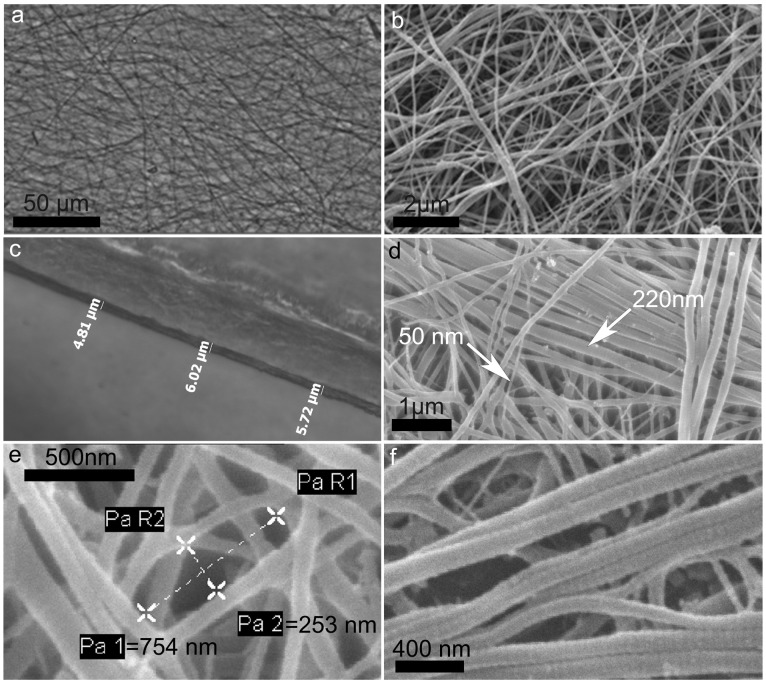
Sea urchin collagen matrix (SCM). (**a**) Light microscopy (LM). After the treatment with Tx-100, collagen fibrils resulted homogeneously distributed on the plastic surface; (**b**) Scanning Electron Microscope (SEM). No cell debris, skeletal parts or undissociated collagen fibers are visible among fibrils; (**c**) LM. The average thickness of SCM is 5–7 µm. The thickness could slightly vary depending on the different areas of the matrix; (**d**) SEM. Different fibrils with different diameters are present in the SCM; (**e**) SEM. The average porosity of the matrix (mesh of the fibrillar interlace) is around 1–2 μm^2^. Below is an example of interlace area measurement. Pa1 = 753 nm (major length); Pa2 = 253 nm (minor length). The area resulted less than 0.2 µm^2^; (**f**) SEM. Collagen D period is well detectable on collagen fibrils.

The collagen suspension was centrifuged, resuspended in Triton-X-100 0.01%, plated on cell culture dishes and left to dry. This step was necessary to minimize surface tension phenomena and thus produce a quite homogeneous matrix thickness on the plastic dish (Figure 4a,b).

Omission of T-X-100 caused a collagen network deposition confined to the dish borders. 

Collagen matrices needed to be cross-linked in order to have suitable mechanical properties for *in vitro* studies or biomechanical manipulations. The collagen matrix was therefore exposed to EDC/NHS mix solution, which acts as a fibril crosslinking agent. After 4 h of exposure, the collagen film resulted tightly adhering to the plastic surface. No significant detachment phenomena were observed, and the inter-fibrillar crosslink apparently displayed a remarkable resistance, even after numerous washings with different media (see Experimental section).

Among the chemical methods for collagen crosslinking, EDC/NHS appeared to be the best option both for providing mechanical resistance to the matrices and for its biocompatibility, already tested by different authors [3,40,41,42,43].

### 2.3. Characterization of Collagen Matrices 

The sea urchin collagen matrices (hereafter referred as SCMs) were observed by SEM to investigate the ultrastructural characteristics of the fibrils as well as their reciprocal interactions and organization. After preliminary tests to find optimal concentrations, SCMs were prepared in a 24 multi-wells using 300 µL of collagen suspension at the concentrations of 2 mg/mL. Values below these limits barely produced a complete and homogeneous covering of the surface. The so produced SCM had an average thickness of 5–7 µm. (Figure 4c).

SEM and light microscopy observations showed a randomly distributed fibril pattern and confirmed the absence of undesired aggregates (cell debris, skeletal parts or undissociated collagen fibers) between the fibrils (Figure 4a,b). Fibril diameter ranged between 25 nm and 300 nm (Figure 4d) which corresponds to what has been reported for mammalian fibrils [48] as well as for the PM [10]. Sea urchin collagen denaturation temperature is 27 °C in *Strongylocentrotus purpuratus* [49]. Nevertheless, despite the step at 37 °C, the experimental protocol did not influence the final chance to get a matrix where fibrils maintained their integrity and could be efficiently cross-linked. 

The SCM “porosity” (mesh of the fibrillar interlace) was maximum 1–2 μm^2^ on the superficial layer (Figure 4e). These values are referred to the dry matrix, observed under SEM high vacuum conditions, and might be slightly higher in wet conditions (as in cell culture studies or in vivo applications). Empirical and preliminary calculation of the swelling ratio indicated that the wet matrix increased its thickness of about 30%. Even considering this swelling variation, the density of collagen matrix is such that mammalian cells could hardly penetrate it and only surface adhesion can be expected.

The D period of collagen fibrils could be clearly observed both after collagen extraction (Figure 2d) and matrix preparation (Figure 4f). This further indicates that the overall protocol did not affect fibril structural integrity.

### 2.4. Biomechanics

Creep (isotonic) and force-extension (isometric) tests were specifically designed and employed to mechanically test the SCM. 

#### 2.4.1. Creep Tests

Maximum stress and viscosity of the SCM were calculated in hydrated conditions (Leibovitz cell culture medium) in order to more closely mimic physiological in vitro or in vivo applications. The initial load (39.57 g) was gradually increased until complete rupture of the collagen scaffold. The breaking load was 17 ± 2.8 MPa (mean ± SD) with minimum and maximum registered values of 13.97 MPa and 24.56 MPa, respectively. 

Collagenous samples, when subjected to a tensile stress, underwent a typical elongation pattern that consisted of three main phases: a primary phase immediately after load application, in which the collagen fibrils of the sample rearranged rapidly, resulting in a substantial sample elongation; a secondary phase in which the extension rate tended to stabilize and in which the viscosity was calculated; a final phase, where the sample was no longer able to support the load and broke. Our results showed that the SCM had generally a very fast primary phase (<2 s) suggesting that the orientation of the collagen fibrils was very rapid soon after the stress was applied. This implies that EDC/NHS crosslinking does not block fibrils in a strictly fixed disposition, thus allowing a certain level of structural matrix reorganization. The tests showed a relatively high variability in the mechanical tensile resistance. 7/36 samples underwent rupture few seconds after starting the test (tensile stress at start was 14–17 MPa), whereas 15/36 samples ruptured within 3 min from the application of the tensile stress. This implies that most of the samples were not able to resist for a long period to a single applied stress of 14–17 MPa. On the other hand, 14/36 samples needed multiple and progressive loads additions before rupture. In this latter case we could calculate the viscosity of the sample in a range between 14 MPa and 24 MPa. Overall, the mean final viscosity of all the tested SCM was 60.98 ± 52.07 GPa·s. This value is much higher than that measured in most fresh tissues of sea urchins and other echinoderms (crinoids, sea cucumbers, *etc*.) as well as in mammalian tissues (human tendons) (Table 1).

**Table 1:** Viscosity of different collagenous tissues. Values are reported as mean ± SD or range.

Structure	Viscosity (MPa·s)	References
Sea urchin compass depressor ligament (*P. lividus*)	560.6 ± 364.7	[50]
Sea urchin spine joint ligament *(Diadema setosum)*	20–5860	[51]
Sea cucumber dermis (*Stichopus japonicus*)	3.0 ± 5.6	[52]
Sea cucumber dermis (*Holothuria leucospita*)	11	[52]
Sea cucumber dermis (*Actinopyga echinites*)	100	[52]
Sea cucumber dermis (*Thyone inermis*)	5100	[53]
Brittle star intervertebral ligament *(Ophiocomina nigra)*	2260 ± 1940	[54]
Feather star stalk (*Cenocrinus asterius*)	16,700	[55]
Human patellar tendon	438.13 ± 232.2	[56]

Only the cirrus apparatus of the crinoid *Cenocrinus asterius* has a higher viscosity than that recorded in our samples (Table 1). Noteworthy, soluble porcine collagen films crosslinked with glutaraldehyde displayed a maximum viscosity of 230.53 MPa·s [57]. This result underlines the relevant mechanical resistance to tensile stress exerted by the SCM, which reflects the presence of strong internal interfibrillar bonds. Most of these bonds were likely due to an effective EDC/NHS collagen crosslinking, possibly helped by the GAG-decorated fibrillar structure of the employed collagen.

#### 2.4.2. Force-Extension Tests

SCM were also evaluated in terms of stiffness (Young’s modulus or elastic modulus). This parameter indicates the capability of a material to resist when subjected to forced elongations (or from another point of view predicts how it reacts to a tensile stress) and it depends on the structural characteristics of the material itself. Stiffness is one of the most important parameters for material engineering applications. Higher stiffness implies less elastic deformation magnitude in response to mechanical stresses and higher energy storage without plastic deformation. 

In our tests the mean calculated stiffness was 146 ± 48 MPa (range: 91–206 MPa) and the mean tensile strength (tension before rupture) was 44.58 ± 9.56 MPa (range 32.85–66.19 MPa) whereas mean tensile strain (relative elongation before rupture) was 32.3% ± 5.8%.

The stiffness of native collagenous tissues/substrates reported in literature is highly variable (Table 2), covering a range from kPa (soluble collagen substrates) to GPa (bones).

Our results, summarized in Table 3, indicate that, even in wet (physiological) conditions, the SCM is a highly resistant material, particularly to uniaxial tensions, with stiffness values in the range of mammalian Achille’s tendons or skin (Table 2). Furthermore, this is an extremely high value if compared to most of the commonly used cell culture collagen substrates, which usually are in an acid-solubilized jelly form. As previously underlined, acid-treatment can cause a partially irreversible denaturation of the collagen molecules, which therefore might reduce its natural and intrinsic mechanical resistance. 

**Table 2:** Stiffness values of different collagenous structures. Values are reported as mean ± SD or range.

Structure	Stiffness (Elastic or Young’s Modulus)	References
Sea urchin compass depressor ligament (*P. lividus*)	16.65 ± 8.93 (Mpa)	[50]
Sea urchin spine catch apparatus (*Anthocidaris crassispina*)	90 ± 0.87 (Mpa)	[58]
Sea cucumber dermis (*Actinopyga mauritiana)*	1 (Mpa)	[59]
Sea cucumber single native collagen fibril (*C. frondosa*)	1–2 (Gpa)	[36]
Bovine single collagen fibril	0.2–0.8 (Gpa)	[60]
Rat Achille’s tendon (*Rattus norvegicus*)	310 (Mpa)	[61]
Pig liver	6.9–34.7 (kPa)	[62]
Human skin	98.97 ± 97 (Mpa)	[63]
Human cornea	0.3–7 (Mpa)	[64]
Human articular cartilage (hip joints)	1.816 ± 0.868 (Mpa)	[65]
Human cortical bone (femoral diaphysis)	17.9 (Gpa)	[65]
Bovine Trabecular bone material	0.76 ± 0.39 (Gpa)	[66]
EDC crosslinked bovine collagen	31 ± 4.4 (Mpa)	[67]
Soluble rat tail collagen (1–3 mg/mL)	1–28 (kPa)	[68]
PCL/collagen scaffold crosslinked with glutaraldheyde	11 (Mpa)	[69]

**Table 3:** SCM mechanical properties. Values are reported as mean ± SD; *n*: number of samples.

Sea Urchin (*P. lividus*) Collagen Matrices (SCM)		
Mechanical Properties	Mean ± SD	*n*
Viscosity	60.98 ± 52.07 GPa·s	36
Breaking load	17± 2.8 MPa	
Stiffness (Elastic or Young’s modulus)	146 ± 48 MPa	19
Tensile strength	44.58 ± 9.56 MPa	
Tensile strain	32.3% ± 5.8%	

Grover *et al.* [67] showed that substrates prepared from a fibrous bovine collagen suspension, crosslinked by EDC-NHS, displayed a stiffness of 31 ± 4.4 MPa (wet conditions), a nearly 5 folds lower value than that we obtained with SCM which was similarly crosslinked. 

A high elasticity of the SCM was observed by comparing the average thickness before and after the mechanical tests and by analyzing the fracture area (SEM and confocal observations). Indeed, the average thickness of the samples did not significantly vary after the mechanical testing (11.16 ± 3.18 μm before; 10.91 ± 3.18 μm after) and its value was constant along the entire length of the samples. Additionally, the fracture surface appeared quite sharp confirming the absence of a plastic deformation.

### 2.5. In Vitro Biocompatibility

**Figure 5:**
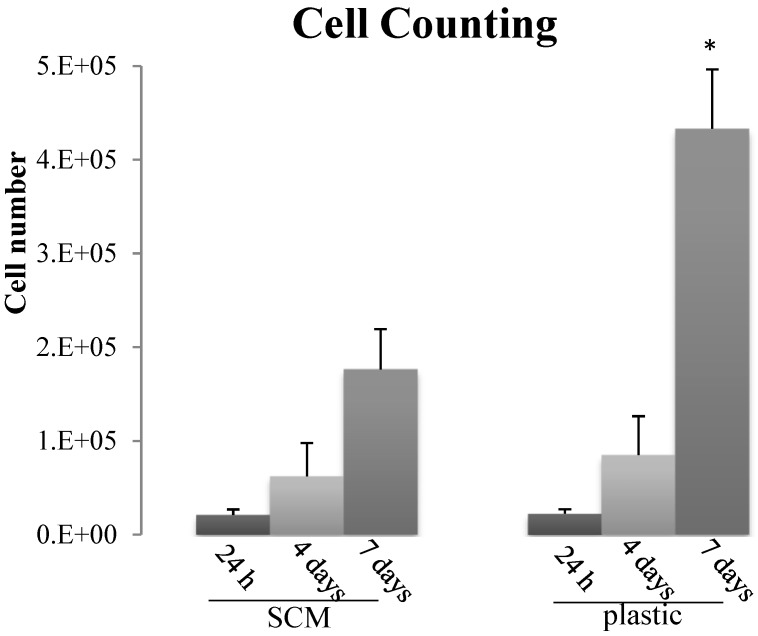
Cell counting of mesenchymal stromal cells (MSCs) seeded on SCM and on plastic at 24 h, 4 and 7 days. Each histogram represents the mean ± SD of four experiments; * *p* < 0.05 plastic *versus* SCM.

**Figure 6:**
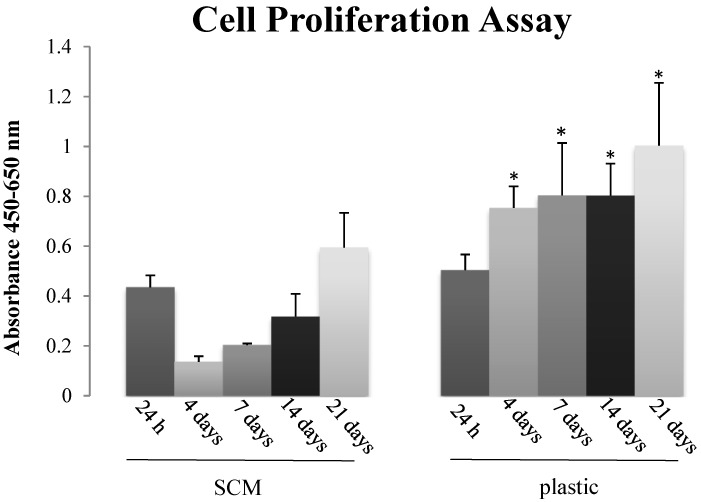
Cell proliferation assay of MSCs seeded on SCM and plastic using XTT Elisa Kit (Roche) at 24 h, 4, 7, 14, 21 days. Each histogram represents the mean ± SD of four experiments; * *p* < 0.05 plastic *versus* SCM.

Preliminary biocompatibility tests were performed to assess mammalian cell behavior when in contact with the SCM; indeed, since these substrates might be used for tissue engineering and regenerative medicine purposes, a first fundamental requirement is the lack of cytotoxicity. Having in mind these potential applications we decided to focus on primary stem cell cultures rather than well established cell lines: mesenchymal stem cells (MSCs) isolated from peripheral blood of horses were therefore grown on SCM to check its suitability as cell substrate. Overall, both cell counting and Cell proliferation Assay (XTT) indicated that the SCM are not toxic for MSCs: despite the lower absolute values than on plastic, cells adhered, survived and in the medium-long time (7–21 days) they also increased in number (Figure 5 and Figure 6), thus suggesting an active proliferation.

The initial decrease of MSCs proliferation on the sea urchin collagen observed in the XTT assay (Figure 6) might suggest a first “adaptation phase” encountered by the cells on the substrate (structurally very different from the flat homogeneous plastic dish); even if present (this was not observed in cell counting tests), this initial phase did not affect the following constant cell growth which led to a subsequent 3 folds increase (Figure 6). In progress studies will help to elucidate this cell behavior and to understand in detail if the SCM influences the cell cycle or the differentiation pathway.

## 3. Experimental Section

Specimens of *Paracentrotus lividus* were collected in Paraggi (“Area Marina Protetta di Portofino”) on the Ligurian coast of Italy. Once transported to the Department of Biosciences (Milan), animals were immediately dissected and their oral halves were stored at −20 °C.

### 3.1. Extraction of Collagen from the PM of P. lividus 

PMs were dissected on ice from frozen oral halves, minced, left in an hypotonic buffer (10 mM Tris, 0.1% EDTA) for 12 h (RT) and rinsed with a decellularizing solution (10 mM Tris, 0.1% SDS) for 12 h (RT). After several and careful washings in PBS, the solution was replaced by a disaggregating solution (0.5 M NaCl, 0.1 M Tris-HCl pH 8.0, 0.1M β-mercapto-ethanol, 0.05 M EDTA-Na) according to Matsumura [18]. The suspension was then filtered and dialyzed against 0.5 M EDTA-Na solution (pH 8.0) for 3 h (RT) and successively against distilled water overnight (RT). Long term storage of the obtained collagen suspension was performed by freezing 1.5 mL aliquots at −20 °C or drying them (37 °C) in silicone molds. These samples were then used for production of either cell culture substrates or samples for mechanical testing (see below).

### 3.2. Ultrastructural Analysis of Isolated Collagen Fibrils

A 50 µL drop of fibril suspension was placed on a 300 mesh copper grid with FORMVAR membrane. After 5 min, the suspension in excess was removed by a filter paper. The grid was stained with uranyl acetate 2% (10 min) and with lead citrate (5 min) and subsequently observed at the transmission electron microscope (TEM JEOL SX 100, Tokyo, Japan).

### 3.3. Cuprolinic Blue Staining for GAG Visualization in Isolated Collagen Fibrils

The isolated collagen samples were applied to 200 mesh Formvar-coated grids. After 5 min, the suspension in excess was removed by a filter paper. The grids were stained with Cuprolinic blue by exposing them sequentially to the following solutions for the indicated times and number of washings [19]: 500 mM NaC1 (60 s × 1), fixative solution (2.5% glutaraldehyde, 25 mM sodium acetate, 300 mM MgCl_2_, pH 5.6; 60 s × 1), Cuprolinic blue (60 s × 1), fixative solution (30 s × 2), sodium tungstate (60 s × 1), water (30 s × 2), 1% uranyl acetate (60 s × 1), and water (30 s × 2). Isolated and unstained fibrils were used as a control. The grids were then observed at the transmission electron microscope (TEM JEOL SX 100, Tokyo, Japan).

### 3.4. SDS-PAGE Analyses of the Collagen Suspension

A 1 mL of stocked collagen suspension (in distilled water) was centrifuged (10,000× *g*, 45 min, 4 °C), the supernatant was discharged and 1 mL of pepsin (1 mg/mL) in acetic acid 0.5 M was added. Samples were left 48 h (4 °C) on an rotary shaker, centrifuged (17,000× *g*, 1 h, 4 °C) and the supernatant was collected in another tube. NaCl was added to reach a final concentration of 5 M, then samples were left on the rotary shaker overnight to allow collagen precipitation (4 °C). The day after samples were centrifuged (16,000× *g*, 1 h, 4 °C), the supernatant was discharged and 500 μL of distilled water were added. The obtained solution was mixed with sample buffer (2:1), heated at 95 °C for 5 min, and run over a 10% precast gel (Biorad, Hercules, CA, USA) at a constant voltage of 150 V. 10 µL of Precision *Plus Protein*™ Dual Color *Standards* (Biorad, Hercules, CA, USA) were used for molecular weight (MW) determination. The separated proteins were visualized by staining with Coomassie Blue R-250.

### 3.5. Production of SCM for Cell Cultures

The concentration of the stored collagen suspension was calculated from a sub-sample as follows: 500 μL of the suspension were diluted in few millilitres of distilled water and centrifuged at 50× *g* (10 min) to remove possible undissociated material. The supernatant was then centrifuged at 2000× *g* (20 min) to obtain a pellet of collagen, and resuspended in 500 μL of deionized water. The so obtained collagen suspension was dried in a silicon mold at 37 °C, overnight. The resulting solid sheet of collagen was weighted and the obtained value was used to calculate the original collagen concentration. Once having this information, the remaining collagen suspension was centrifuged at 50× *g* (10 min.) and then at 2000× *g* for 20 min. The pellet was rinsed with 0.01% TritonX-100 in a proper volume to reach 2 mg/L final collagen concentration. 300 μL of collagen suspension were placed in each 24-multiwells dish and left at 37 °C overnight. The so obtained SCM were then exposed to 300 μL EDC/NHS cross-linker solution (EDC 30 mM/NHS 15 mM in MES buffer 100 mM) for 4 h and subsequently washed several times with PBS, distilled water and ethanol 70%. In order to check their suitability (cleanness and homogeneity), the obtained SCMs were observed under the inverted microscope Axiovert 200M (Zeiss, Oberkochen, Germany)—AxioCam HRM HAL 100, equipped with an image acquisition system Axio Vision Rel 4.5. Prior to use, SCM were carefully washed with Leibovitz medium, the same used in the following cell cultures.

### 3.6. Production of SCM for Mechanical Tests 

The protocol’s first steps were the same previously described for cell culture substrates production. Then 800 μL of the 2 mg/mL suspension were placed in a specifically prepared rubber silicone mold (10 mm × 16 mm) and left to dry at 37 °C overnight. Samples were then treated as previously described with EDC/NHS solution and washed with PBS and distilled water. The so obtained SCM were then cut into small strips (2 mm × 10 mm) to be tested for mechanical tests. Four strips were used for thickness evaluation by confocal microscopy (Leica TCSNT confocal laser microscope, Wetzlar, Germany) and/or SEM (LEO-1430, Zeiss, Oberkochen, Germany).). For confocal analysis strips were stained with Sirius red (50 μM in acetic acid 0.5 M) for 20 min, then washed with PBS to remove the residues of the dye and mounted on a glass slide. Thickness of sample for mechanical tests was 11–12 μm.

### 3.7. Scanning Electron Microscopy (SEM)

SCMs were washed in 0.1 M sodium cacodylate buffer overnight and then fixed with 2% glutaraldehyde in 0.1 M cacodylate buffer (2 h, 4 °C). This fixation preceded a secondary fixation with 1% osmium tetroxyde in 0.1 M sodium cacodylate buffer (2 h, RT). After being washed with distilled water, samples were dehydrated with increasing percentages of ethanol (25%, 50%, 70%, 90%, 100%). Absolute ethanol was gradually substituted with HMDS (Hexamethyldisilazane) (25%, 50%, 75%, 100%). After that, SCMs were left to dry on a filter paper. Samples were then mounted on stubs and covered with pure gold (Agar SEM Auto Sputter, Stansted, UK). Finally, samples were observed at the scanning electron microscope (LEO-1430, Zeiss, Oberkochen, Germany).

### 3.8. Mechanical Tests

**Figure 7:**
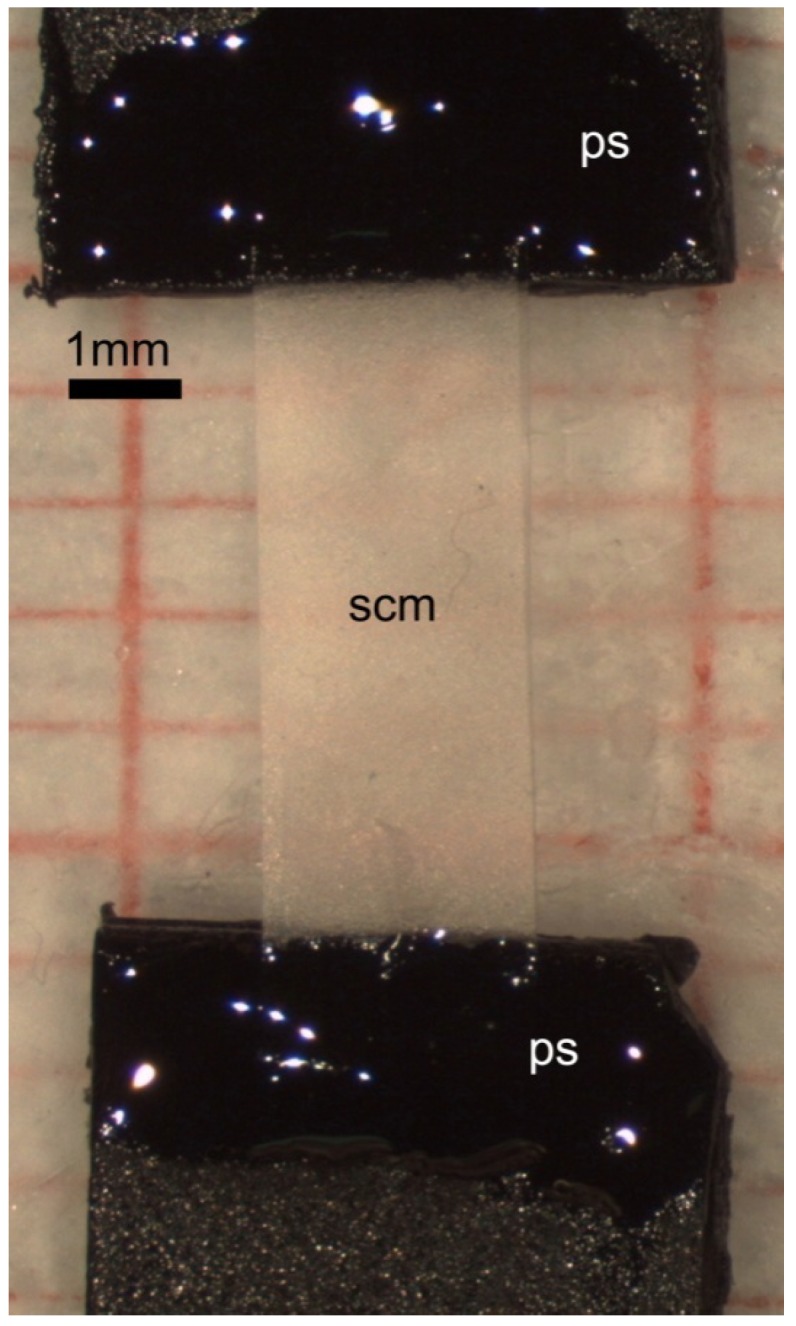
LM. SCM for mechanical test. Plastic support (ps), sea urchin collagen matrix (scm).

The mechanical properties of SCM were investigated under creep (isotonic test) and force-extension (isometric tests) conditions. The experimental apparatus consisted of an isotonic force transducer (Harvard Apparatus, Holliston, MA, USA) or an isometric force transducer (LCM System Ltd., Newport, UK) plugged to a PowerLab 2/26 recorder device (AD Instrument, Dunedin, New Zealand) via pre-amplifier. All the data originated from the mechanical tests were recorded with Labchart 7 software (AD Instrument, Dunedin, New Zealand). Each SCM strip was fixed at the two ends to rigid plastic supports with cyanoacrylate cement (Superattak^®^, Heckel, Düsseldorf, Germany) so that the strip length left between the supports was 6 mm. Each strip was photographed before testing (Figure 7).

In order to mimic as much as possible a physiological condition under mechanical stress all the SCMs were tested in L-15 Leibovitz cell culture medium; samples were immersed and hold in position 5 min before the test started and kept in immersion for all the duration of the experiments.

#### 3.8.1. Creep Tests

Twenty-nine strips were used for creep tests. The upper part of each strip was connected with a stainless steel chain to the lever of the isometric force transducer; the lower part was tightly clamped to a fixed hook. A locking device prevented the elongation of the samples before the start of the test. From the obtained curves we extrapolated the strain rate in order to calculate the viscosities of the samples. The breaking load was the load at which the samples broke. Viscosity values were calculated as follows:

(1) $$\text{Coefficient of viscosity}\;(MPa\cdot s)=\frac{Nominal\;stress}{strain\;rate}$$

(2) $$\text{Nominal stress}\;(N/{mm}^{2})=\frac{Force}{Initial\;CSA\;(sample\;cross\;\sec tion\;area)}$$

(3) $$\text{Strain rate}=\frac{\Delta l}{\Delta t\;(mm/s)}$$

#### 3.8.2. Force-Extension Test

Nineteen strips were used for force-extension tests. The upper part of the strip was attached to the force transducer with a stainless steel chain whereas the lower part was fixed to a hook connected with a micromanipulator. Samples were subjected to elongation of 0.1 mm every 10 s until rupture or until reaching the maximum load of the transducer. The force peaks generated at each elongation step were used to produce a stress-strain curve; the tangent to the steepest straight-line portion of each curve was used to calculate the stiffness of each sample. The breaking strain and breaking stress were obtained from the curves when samples underwent rupture. Stiffness values were calculated as follows:

(4) $$\text{Stiffness (MPa)}=\frac{\Delta \;stress}{\Delta \;strain}$$

(5) $$\Delta \text{Stress (MPa)}=\frac{\Delta F}{CSA}$$

(6) $$\Delta \text{strain}=\frac{\Delta l}{l\;(starting\;sample\;length)}$$

### 3.9. Biocompatibility

#### 3.9.1. Mesenchymal Stromal Cell Cultures

Mesenchymal stromal cells (MSCs) were obtained from equine peripheral blood by gradient separation [70] and they were maintained at 37 °C in an incubator with an atmosphere of humidified air 5% CO_2_ in growth medium.

#### 3.9.2. Cell Counting

The MSCs were seeded at a concentration of 2 × 10^4^/well in 6-wells (plastic wells and wells treated with MCT 5 mg/mL) for 24 h, 4 and 7 days. At different time points the cells were trypsinized and counted with Burker chamber. Four experiments were carried out and for each experiment 6 replicates were performed.

#### 3.9.3. Proliferation Assay

Cell proliferation was evaluated using the Cell Proliferation Kit II (XTT)-base (Roche, Milan, Italy) colorimetric assay. Cells (1 × 10^4^) were grown in 96-well tissue culture plates for 24 h, 4, 7, 14 and 21 days. MSC were grown in plastic wells and in wells plated with SCM. At each time point, cells in selected wells were incubated with the yellow XTT solution for 1 h. After incubation with XTT, the metabolically active cells developed an orange formazan product, which was quantified using an enzyme-linked immunosorbent assay plate reader (Spectra Count, Perkin Elmer, Milan, Italy). The amount of orange formazan produced was directly correlated to the number of living cells. Four experiments were carried out and for each experiment three replicates were performed.

### 3.10. Statistical Analysis

Data are expressed as the mean ± SD. Statistical analysis was performed using the paired Student *t*-test (SPSS software, version 11.0). The level of statistical significance was set at *p* ≤ 0.05.

## 4. Conclusions 

The sea urchin is a well known and common experimental model widely used in basic and applied biology. *P. lividus* is also an edible species, appreciated for the delicacy of its gonads and therefore collected and often cultured in many countries for alimentary purposes. All the non-edible body parts, such as the PM, could be collected and used as a recycled material for producing low-cost collagen substrates.

The protocol here proposed and developed allows us to obtain native collagen fibrils in sufficient amount and purity to produce a cohesive matrix. High resistance to mechanical stress and elasticity are the main viscoelastic properties of the produced SCM, which therefore might be suitably employed for specific tissue engineering applications such as tendon or skin regeneration (Table 1, Table 2 and Table 3). Alternatively, they might be useful as highly resistant dermal stitches for surgical purpose or skin tape for topical applications (lacerations or burns). Furthermore, our results showed that the sea urchin collagen matrices may be successfully seeded with mesenchymal stromal cells isolated from equine blood thus indicating they can be a promising clinical tool for mammalian damaged tissues in regenerative medicine. Further ongoing *in vitro* and *in vivo* tests will help to confirm the SCM biocompatibility. In conclusion, we think that the SCM might provide cells a more biomimetic environment in terms of structure (fibrillar), biochemical composition (collagen) and mechanical characteristics than other existing substrates. 
